# Harnessing Biogenic Silica: Nanoarchitected Pt_3_Pd_1_ on Nettle-Derived N,Si-CQDs for High-Performance Methanol Electrooxidation

**DOI:** 10.3390/nano15201561

**Published:** 2025-10-14

**Authors:** Seden Beyhan

**Affiliations:** Department of Chemistry, Faculty of Science and Letters, Istanbul Technical University, Maslak 34469, Istanbul, Turkey; beyhanse@itu.edu.tr

**Keywords:** electrocatalysts, carbon quantum dot, N-Si-CQDs, biogenic silica, Pt_3_Pd_1_, methanol oxidation

## Abstract

This study introduces nitrogen- and silicon-containing carbon quantum dots (N,Si-CQDs), synthesized hydrothermally from the sustainable bioresource stinging nettle (*Urtica dioica* L.), as chemically active supports for Pt, Pd, and Pt_3_Pd_1_ electrocatalysts. The N,Si-CQDs were characterized by a high concentration of N/O surface functionalities and the presence of biogenic Si. A significant finding is that, with this support, biogenic Si acts as a nucleation template: Pd forms in situ as orthorhombic Pd_9_Si_2_ nanorods alongside spherical particles, whereas Pt predominantly develops as cubic/quasi-cubic crystals. This templating process promotes faceted (cubic) Pt_3_Pd_1_ alloy nanoparticles with robust interfacial contact with the support and a log-normal size distribution (14.2 ± 4.3 nm) on N,Si-CQDs (4.7 ± 1.4 nm). This configuration enhanced the electrochemically active surface area to 181 m^2^ gPt^−1^, significantly exceeding those of commercial Pt_1_Pd_1_/XC-72 (27.7 m^2^ gPt^−1^) and monometallic Pt/N,Si-CQDs (14.3 m^2^ gPt^−1^). Consequently, the catalyst demonstrated superior methanol oxidation performance, evidenced by a low onset potential (0.17 V), approximately 10-fold higher mass activity compared to Pt_1_Pd_1_/XC-72, and 53% activity retention after a 16 h accelerated durability test. The enhanced performance is attributed to the strong nanoparticle anchoring by N,Si-CQDs, the bifunctional/ligand effects of the Pt–Pd alloy that improve CO tolerance, and the templating role of biogenic Si.

## 1. Introduction

Advancing sustainable energy technologies is a critical objective of contemporary research. Electrochemical energy conversion systems, particularly fuel cells, are characterized by their high efficiency and minimal environmental impact. Among alcohol-fed systems, direct methanol fuel cells (DMFCs) are notable for their high energy density and the convenience of liquid fuel logistics, rendering them particularly appealing for use in portable electronic devices [1,2,3,4]. Despite the potential of fuel cells, their performance is frequently limited by electrode kinetics, particularly the oxygen reduction reaction (ORR) at the cathode and, in the case of DMFCs, the methanol oxidation reaction (MOR) at the anode. Additionally, the poisoning of active sites by intermediates, such as carbon monoxide (CO), poses a significant challenge. Although Pt-based materials continue to serve as the standard for MOR catalysts, the high cost of Pt and its inherent vulnerability to CO poisoning remain significant barriers to their widespread adoption [2,3].

To address these limitations, two complementary strategies have been developed: (i) alloying Pt with a secondary metal to modify the surface chemistry and (ii) designing advanced supports to stabilize and activate nanoparticles [4,5]. Among the alloy combinations, Pt–Pd is particularly effective. Pd facilitates a bifunctional mechanism for oxidative CO removal and induces electronic (ligand) effects through intermetallic charge transfer, thereby weakening the Pt–CO bond and enhancing the MOR [2]. The performance is significantly influenced by the composition and nanostructure, with Pt_3_Pd_1_ being identified as a nearly optimal formulation. Importantly, Pt_3_Pd_1_ supported on ceria-modified carbon (CeO_2_/C) demonstrates enhanced activity and durability, which can be attributed to the synergistic interactions between the alloy nanoparticles and the redox-active oxide support [6]. In addition to alloy selection, the systematic engineering of catalysts at the nanoscale, referred to as nanoarchitecture, is crucial for optimizing the electrochemically accessible surface area and metal utilization. This process involves the intentional control of the nanoparticle size, shape, dispersion, and interfacial chemistry to ensure robust anchoring and favorable electronic coupling [7,8,9].

In this framework, the performance of electrocatalysts is contingent not only on the structure of the active nanoparticles but also on the properties of the support. This support is now recognized as an active component rather than merely an inert carrier. An effective support must disperse nanoparticles across a large surface area, provide high electrical conductivity, and maintain stability under harsh conditions, among other requirements. Conversely, amorphous carbon blacks (e.g., Vulcan XC-72) are susceptible to electrochemical corrosion, which facilitates particle detachment/agglomeration and results in a loss of the electrochemically active area. Next-generation carbon materials, such as graphene, carbon nanotubes, and carbon quantum dots (CQDs), have been investigated owing to their high accessible surface area, excellent conductivity, and adjustable surface chemistry [10,11,12,13,14]. Notably, CQDs exhibit zero-dimensional structures characterized by abundant edge defects and surface functional groups (e.g., carboxyl, hydroxyl, and amine), which provide robust anchoring and nucleation sites, thereby facilitating interfacial chemistry that mitigates the degradation [10].

Simultaneous heteroatom doping of CQDs with nitrogen, boron and phosphorus enhances metal–support interactions and modulates the electronic structure, thereby presenting a promising approach for the co-optimization of both activity and durability [11,12,13,14]. Nevertheless, the utilization of biomass precursors that inherently contain these heteroatoms has been relatively neglected. The prevailing perspective often regards these intrinsic elements as “impurities,” disregarding their potential active roles in the nucleation of nanoparticles and alloy formation. Similarly, supports are frequently perceived as passive stabilizers, with their potential function as active components that direct nucleation and phase evolution remaining unexplored. Consequently, this study aims to elucidate the influence of intrinsic heteroatoms on the morphological and electronic characteristics of CQDs and the impact of these characteristics on the catalytic performance of supported nanoalloys.

In this study, stinging nettle (*Urtica dioica*) was selected as the sole biomass precursor for synthesizing N,Si-containing CQDs. Nettles inherently provide nitrogen and accumulate biogenic amorphous silica (phytoliths), facilitating in situ heteroatom incorporation. Consequently, the electron-donating properties of nitrogen, which enhance conductivity [12,13], and the role of silicon in creating structural defects that serve as active sites, along with its oxyphilic nature that aids in the activation of oxygenated intermediates [15], are effectively unified on a single platform. This integration is anticipated to enhance oxidative processes such as the MOR. Therefore, this biomass-derived multi-heteroatom strategy delineates a direct pathway to chemically active supports that integrate structural, interfacial, and electronic tuning, thereby laying the foundation for next-generation Pt–Pd electrocatalysts.

The primary aim of this study was to synthesize highly dispersed monometallic Pt and Pd, as well as bimetallic Pt_3_Pd_1_, on nettle-derived N,Si-CQDs, and to elucidate the structure–performance relationships. To achieve this, comprehensive characterization was performed to ascertain the morphological, structural, and surface chemical properties, which were subsequently correlated with the MOR activity in comparison with a commercial reference, Pt_1_Pd_1_ on Vulcan XC-72. The findings reveal that biogenic Si inherent to nettle-derived CQDs plays a pivotal role in directing the formation of highly dispersed nanoalloys, enabling up to a ten-fold enhancement in the MOR activity relative to the commercial benchmark. These results suggest that the combined effects of alloy architecture, CQD surface chemistry, and strong interfacial interactions underpin the superior performance, positioning Pt_3_Pd_1_/N,Si-CQDs as a promising candidate for next-generation electrocatalysts.

## 2. Materials and Methods

### 2.1. Materials

Hexachloroplatinic acid hexahydrate (H_2_PtCl_6_·6H_2_O) and potassium tetrachloropalladate (II) (K_2_PdCl_4_) were purchased from Alfa Aesar (Heysham, UK). Nafion^®^ solution (5 wt.%), sulfuric acid (H_2_SO_4_, 95–97%), methanol (CH_3_OH, 99.8%), and absolute ethanol were purchased from Sigma-Aldrich (St. Louis, MO, USA). Ultrapure water (18.2 MΩ·cm, ELGA Purelab Option-Q,High Wycombe, UK), was used in all experiments. Stinging nettle (*Urtica dioica* L.) leaves were collected from the Ayazaga Campus of Istanbul Technical University, washed, and oven-dried at 50 °C for 24 h. A commercial 10 wt.% Pt_1_Pd_1_/Vulcan XC72 catalyst (10 wt.% total metal) was purchased from Fuel Cell Store (College Station, TX, USA) and used as a reference.

### 2.2. Synthesis of N,Si-CQDs

N,Si-CQDs were synthesized from stinging nettle leaves via a hydrothermal route. In a typical procedure, 7.5 g of dried, powdered leaves (sieved through a 250 µm mesh) were dispersed in 150 mL of ultrapure water and transferred to a 250 mL PPL-lined stainless-steel autoclave. The vessel was sealed and maintained at 225 °C for 24 h (autogenous pressure). After cooling to room temperature, the resulting brown dispersion was centrifuged at 9000 rpm for 10 min to remove coarse particulates, and the clear supernatant was passed through a 0.22 µm syringe filter (mixed cellulose ester, MCE). The filtrate was stored at 4 °C until use.

For characterizations requiring a solid sample, an aliquot of the N,Si-CQDs dispersion was freeze-dried (lyophilized) for −65 °C for 72 h (Biobase, Jinan, Shandong, China), yielding a waxy solid. The concentration of the aqueous N,Si-CQDs dispersion was 0.017 g mL^−1^.

### 2.3. Preparation of Pt-, Pd- and Pt_3_Pd_1_/N,Si-CQDs

All electrocatalysts were synthesized via a hydrothermal reduction method, aiming for a total metal loading of 5 wt.% on the N,Si-CQDs support. Prior to utilization, the aqueous N,Si-CQDs dispersion, stored at 4 °C, was filtered through a 0.22 µm syringe filter (MCE). For the bimetallic Pt3Pd1/N,Si-CQDs, stoichiometric quantities of H2PtCl6⋅6H2O and K2PdCl4 were measured to achieve a 3:1 Pt:Pd atomic ratio and were subsequently dissolved in the filtered N,Si-CQDs. For the monometallic Pt/N,Si-CQDs and Pd/N,Si-CQDs catalysts, only the respective precursor salts were employed. The precursors were allowed to dissolve completely under magnetic stirring at 300 rpm for 30 min. The resulting homogeneous mixture was transferred to a 250 mL PPL-lined stainless-steel autoclave, sealed, and heated at 225 °C for 24 h. Following cooling to room temperature, the black precipitate was collected via centrifugation at 6000 rpm for 10 min. The solids were purified through two washing cycles with ultrapure water, followed by two cycles with ethanol; each cycle involved resuspension, centrifugation at 6000 rpm for 10 min, and decantation of the supernatant. Finally, the purified catalyst was dried in an oven at 60 °C for 12 h (Figure S1).

### 2.4. Physical and Chemical Characterization

The structural, morphological, and optical characteristics of the synthesized N-Si-CQDs and supported catalysts were comprehensively characterized. Ultraviolet-Visible (UV–Vis) absorption spectra were recorded in the range of 200–700 nm using a double-beam spectrophotometer (UV-6300PC, VWR International, Radnor, PA, USA). Photoluminescence (PL) measurements were conducted using a fluorescence spectrometer (Cary Eclipse, Agilent Technologies, Santa Clara, CA, USA). Steady-state emission spectra were obtained at an excitation wavelength of 350 nm, with excitation/emission slit widths of 5 nm and a scan rate of 600 nm·min^−1^. Additionally, excitation–emission matrix (EEM) maps were acquired by scanning the excitation from 280 to 350 nm and the emission from 360 to 600 nm. Surface functional groups were identified using Attenuated Total Reflection-Fourier Transform Infrared (ATR-FTIR) spectroscopy with a diamond crystal (Nicolet 380, Thermo Fisher, Waltham, MA, USA). Spectra were acquired from 4000 to 500 cm^−1^ at a resolution of 4 cm^−1^ with 32 scans per sample. The crystal structure and phase composition were examined via X-ray diffraction (XRD; D-Max 2200, Rigaku, Tokyo, Japan) using Cu Kα radiation (λ = 1.5406 Å). Data were collected at room temperature over a 2θ range of 10–80°, with a step size of 0.05° and step time of 110 s per step. The surface elemental composition and chemical states were analyzed by X-ray photoelectron spectroscopy (XPS; K-Alpha, Thermo Fisher Scientific, Waltham, MA, USA) with monochromatic Al Kα radiation (1486.3 eV); a 400 µm X-ray spot and pass energy of 50.0 eV were employed. The spectra were calibrated to the C 1s peak at 284.5 eV, and peak deconvolution was performed using Avantage 5.9 software. The morphology and particle size were evaluated using transmission electron microscopy (TEM; HT-7700, Hitachi, Tokyo, Japan) equipped with a LaB_6_ electron source and operated at 120 kV. For TEM sample preparation, the catalyst was ultrasonically dispersed in ethanol, a droplet was deposited onto a 200-mesh copper grid, and the solvent was allowed to evaporate under ambient conditions. For size-distribution analysis by TEM, all samples were analyzed by measuring at least 200 primary particles using ImageJ v1.54g (NIH, Washiontan, DC, USA) and number-weighted size distributions were reported.

### 2.5. Electrochemical Measurements

All electrochemical measurements were conducted at ambient temperature utilizing a potentiostat/galvanostat (VoltaLab PGZ402, Radiometer Analytical, Lyon, France) within a standard three-electrode configuration. The catalyst ink was formulated by ultrasonically dispersing 3 mg of catalyst powder in 60 µL of a 5 wt.% Nafion^®^ solution (Sigma-Aldrich, St. Louis, MA, USA) and 300 µL of ultrapure water. A 3 µL aliquot of the homogeneous ink was deposited onto a glassy carbon electrode (GCE, geometric area 0.071 cm^2^) that had been previously polished with 0.05 µm alumina and thoroughly cleaned. Following drying under ambient conditions, the total metal (Pt + Pd) loading was determined to be 1.25 µg per electrode (equivalent to 17.6 µg cm^−2^ on the 0.071 cm^2^ GCE).

The electrochemical cell comprised a catalyst-modified GCE as the working electrode, a graphite rod as the counter electrode, and an Ag/AgCl (saturated KCl) as the reference electrode. The electrolytes (0.5 M H_2_SO_4_ and 0.5 M H_2_SO_4_ + 0.5 M CH_3_OH) were saturated with N_2_ prior to the measurements. Each electrochemical test was conducted in triplicate using freshly prepared working electrodes. Prior to MOR measurements, 30 consecutive cyclic voltammetry (CV) cycles were executed in N_2_-saturated 0.5 M H_2_SO_4_ at 50 mV s^−1^ to attain a steady-state active surface and ensure electrode stabilization.

Cyclic voltammetry was recorded between −0.2 and +1.0 V vs. Ag/AgCl (scan rate 50 mV s^−1^). Mass activity (MA) was defined as the current at the forward peak potential (E_P_) normalized to the total metal loading of the deposited ink and reported in A/g_metal_. The electrochemical surface area (ECSA) was calculated from the hydrogen adsorption/desorption charge (H_upd_ region) in 0.5 M H_2_SO_4_, following a double-layer correction. This calculation utilized a value of 210 μC cm^−2^ for one monolayer of hydrogen on polycrystalline Pt, and the results were normalized by Pt loading, expressed as m^2^/g_Pt_ [16,17]. For the Pt–Pd samples, the ECSA pertains exclusively to Pt. The corresponding specific activity (SA) was obtained as SA = MA/S_ECSA_ [18], where S_ECSA_ is the electrochemically active surface area determined from the H_upd_ region in 0.5 M H_2_SO_4_ and normalized to Pt and expressed in mA/cm^2^. Chronoamperometry (CA) was performed in a methanol-containing electrolyte at 0.50 V for 1800 s. Accelerated durability test (ADT) was conducted in the same electrolyte by applying 1200 potential cycles between −0.2 and +1.0 V at 50 mV s^−1^ (total duration ~16 h).

## 3. Results

### 3.1. Characterization of N,Si-CQDs

**Figure 1:**
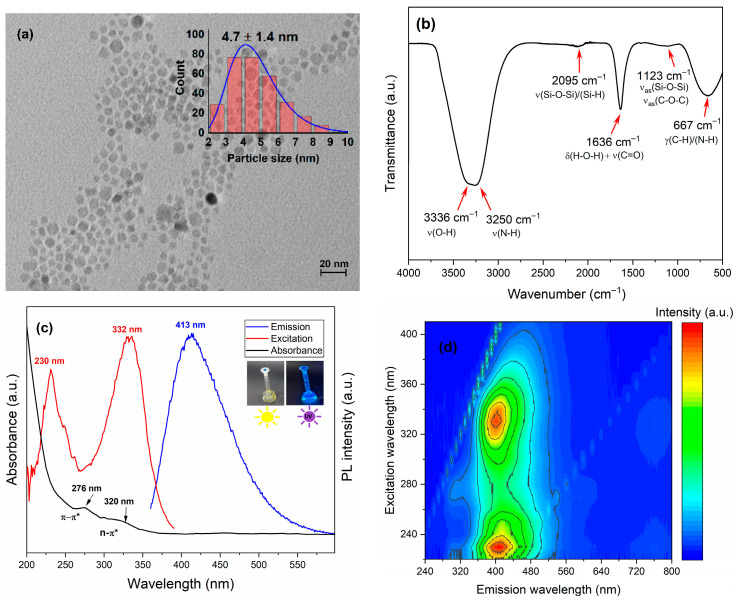
Structural and optical characterization of N,Si–CQDs. (**a**) TEM micrograph accompanied by a size histogram (scale bar 20 nm), (**b**) FTIR spectrum displaying characteristic bands, (**c**) UV–vis absorbance (black), PL excitation (red), and emission (blue) spectra; inset: photographs under daylight and 365 nm UV light. (**d**) Three-dimensional excitation–emission contour map.

The morphology and particle size were evaluated using TEM (Figure 1a). The images depict well-dispersed quasi-spherical dots with an average diameter of less than 5 nm and minimal aggregation. Surface functional groups were identified using FTIR (Figure 1b). The spectrum displays a broad envelope between 3500 and 3000 cm^−1^ attributed to O-H stretching vibrations of hydroxyl groups, overlapping with N-H bands [19]. A weak feature at 2095 cm^−1^ potentially indicates the presence of Si–H and/or conjugated C≡C species. The pronounced feature at 1636 cm^−1^ is mainly assigned to the H–O–H bending of adsorbed water, with additional contributions from ν(C=C) (aromatic domains) and ν(C=O) (amide-I/carboxylate), confirming the presence of hydroxyl and carboxyl surface groups [20]. A peak at 1123 cm^−1^ was assigned to Si–O–Si and/or C–O–C stretching [21,22,23], while a low-frequency band at 667 cm^−1^ was attributed to out-of-plane bending vibrations of aromatic C-H and N-H bonds. Collectively, these signatures confirm the presence of N and Si heteroatoms, together with oxygenated functionalities, indicating effective surface passivation.

The physicochemical properties of carbon dots synthesized from stinging nettle leaves were characterized to elucidate their function as catalyst supports. The analyses confirmed that the synthesis yielded a multifunctional naturally heteroatom-enrichedC-N-Si composite, rather than a mere carbon support. The optical properties were examined using UV-Vis and PL spectroscopy (Figure 1c). The UV-Vis spectrum displays a distinct absorption peak at 276 nm and a shoulder near ~320 nm. The peak at 276 nm is attributed to the π→π* transitions of aromatic sp^2^-C=C bonds, while the shoulder at ~320 nm arises from n→π* transitions of C=O/C=N groups and the dopant-induced surface states [24]. As a consequence of this absorption, the excitation of the material at 332 nm generated an intense blue emission band centered at ~413 nm. This results in a large Stokes shift of ~81 nm between absorption and emission, indicating that radiative recombination occurs via stabilized emissive surface states associated with the N/Si dopants and C=O/C=N groups. The 3D EEM map (Figure 1d) confirms a ~230 nm domain (core π→π*) and a dominant excitation band at ~330 nm associated with N/Si-related emissive centers. The modest excitation dependence of the emission peak suggests the prevalence of a single, dominant emissive state [25]. This, in turn, is indicative of effective surface passivation, which explains the high photoluminescence intensity (the red-yellow regions in the map).

**Figure 2:**
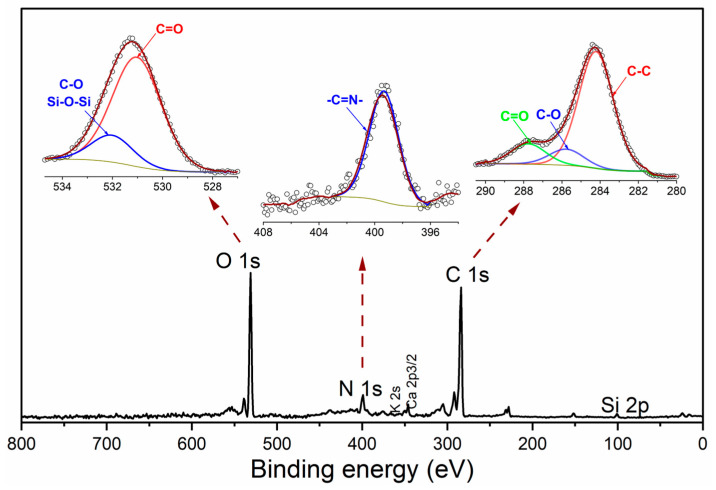
Survey scan and high resolution XPS spectra for N,Si-CQDs.

XPS was used to conduct a comprehensive quantitative analysis of the surface elemental composition and chemical states of the samples (Figure 2). The survey scan ) confirmed the presence of C (66.42 at.%), O (21.37 at.%), N (7.24 at.%), and Si (2.29 at.%), along with minerals such as Ca (1.05 at.%) and K (1.64 at.%). The high-resolution C 1s spectrum was deconvoluted into three primary components: C–C/C=C at 284.2 eV (76.19 at.%), C–N/C–O at 285.9 eV (10.15 at.%), and C=O/O–C=O at 287.8 eV (13.26 at.%) [26]. These binding energies fall within the typical range reported for CQDs (284.5–288.5 eV) [27,28], confirming the validity of the fitted components. Minor variations may originate from heteroatom (N, Si) doping and the biogenic carbon source. The N 1s envelope exhibits a broad peak centered at 399.3 eV, predominantly characterized by pyrrolic/imine-N (-C=N-) with a minor contribution from pyridinic-N [19,26]. These nitrogen functionalities serve as anchoring or coordination sites (Pt–N/Pd–N), thereby enhancing the metal–support interactions (MSI) and facilitating electron transfer. The O 1s spectrum was resolved into two main peaks, C=O at ~531.0 eV (82.70 at.%) and C–O/Si–O–Si at ~532.0 eV (17.30 at.%) [26], in strong agreement with the 1123 cm^−1^ FTIR band. In summary, the combined data demonstrate that N-Si co-doping and oxygenated surface groups stabilize the sp^2^ carbon nanodomains and surface states. This unique structure, derived from a sustainable precursor, enables bright blue photoluminescence and provides chemically active anchoring sites that are ideal for prospective metal–support interactions in catalysis.

### 3.2. Characterization of Pt-, Pd-, and Pt_3_Pd_1_/N,Si-CQDs

**Figure 3:**
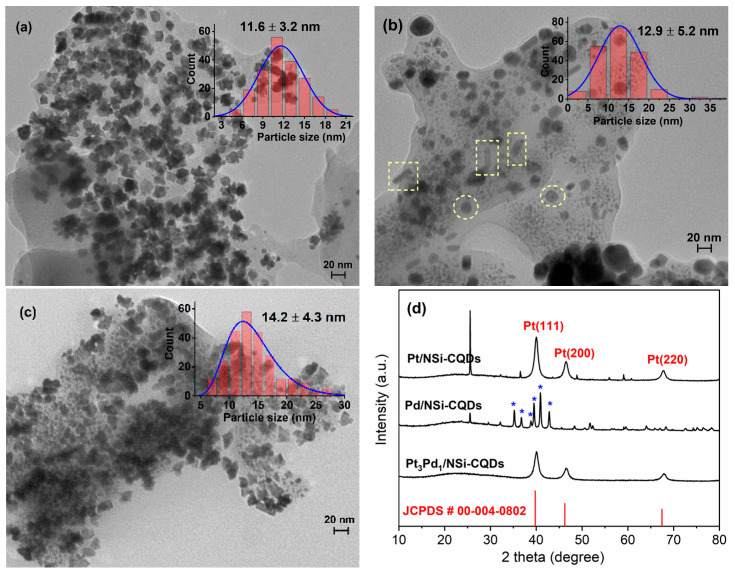
Representative TEM micrographs of (**a**) Pt/N,Si-CQDs, (**b**) Pd/N,Si-CQDs, (**c**) Pt_3_Pd_1_/N,Si-CQDs (scale bars: 20 nm) with particle-size histograms (mean ± SD), and (**d**) XRD patterns of catalysts. The yellow dashed boxes and circles in (b) highlight the rod-like elongated nanostructures and spherical particles that are characteristic of Pd/N,Si-CQDs. Asterisks (*) in (**d**) denote reflections corresponding to orthorhombic Pd_9_Si_2_ (JCPDS #00-041-1102).

The TEM micrographs of the Pt/N,Si-CQDs, Pd/N,Si-CQDs, and Pt_3_Pd_1_/N,Si-CQDs samples revealed that the metal nanoparticles were uniformly distributed on the N,Si-CQDs support, with no evident agglomeration (Figure 3a–c). The particle sizes (mean ± SD) were 11.6 ± 3.2 nm for Pt/N,Si-CQDs, 12.9 ± 5.2 nm for Pd/N,Si-CQDs, and 14.2 ± 4.3 nm for Pt_3_Pd_1_/N,Si-CQDs. Pt/N,Si-CQDs was characterized by distinctly cubic/cuboidal particles, whereas Pt_3_Pd_1_/N,Si-CQDs displayed a dense and uniform population of quasi-spherical nanoparticles covering the support. In Pd/N,Si-CQDs, notably—and unique to this sample—rod-like elongated nanostructures appeared alongside perfectly spherical particles; both motifs are highlighted with yellow dashed outlines in Figure 3b and are not prominently observed in the other catalysts. Statistically, Pt/N,Si-CQDs and Pd/N,Si-CQDs follow normal size distributions, while Pt_3_Pd_1_/N,Si-CQDs exhibits a log-normal distribution; a similar log-normal behavior is also observed for the bare N,Si-CQDs sample (Figure 1a).

The XRD patterns corroborate the TEM observations at the structural level (Figure 3d). For Pt/N,Si-CQDs, the primary reflections of fcc Pt are discernible; for instance, the (111) peak at 2θ = 39.984° and the higher-angle (220) peak at ≈67.734° align with JCPDS 00-004-0802 and are consistent with the cubic morphology, indicating a significant contribution from the {100} facets [29]. The corresponding interplanar spacings, calculated from Bragg’s law, are 0.225 nm for (111) and 0.138 nm for (220), in good agreement with the standard values of fcc Pt. In Pt_3_Pd_1_/N,Si-CQDs, all metallic reflections formed a single fcc set situated between the Pt and Pd positions; the (111) peak at 40.048° and the (220) peak at ≈ 67.873° are indicative of a substitutional Pt–Pd solid-solution alloy. For comparison, typical Cu Kα reference positions for fcc Pd are (111) ≈ 40.12°, (200) ≈ 46.66°, (220) ≈ 68.12°, and (311) ≈ 82.10° (ICDD/JCPDS 00-046-1043). In the Pd/N,Si-CQDs sample, additional reflections (marked in blue in Figure 3d) emerged that could not be attributed solely to fcc Pd and were identified as palladium silicides, mainly Pd_9_Si_2_ (JCPDS 00-041-1102), based on ICDD reference data. This interpretation aligns with the established tendency of Pd and Si to interdiffuse and form Pd–Si silicides, including colloidal Pd_2_Si, as confirmed by XRD [30]. When considered alongside the nanorod motif observed exclusively in this sample by TEM, these extra peaks suggest the presence of an additional palladium silicide phase (Pd_9_Si_2_) and the onset of direction-dependent (anisotropic) growth. These observations are consistent with kinetically driven anisotropic growth of twinned Pd nanostructures that expose high-index facets, as previously reported by Wang et al. [31]. In all patterns, the broad, weak band at ≈25° corresponds to the graphitic (002) contribution of the N,Si-CQDs support. Figure 3 collectively illustrates the formation of cubic Pt nanoparticles enriched in the {100} facets for Pt/N,Si-CQDs, the presence of both icosahedral particles and nanorods (exhibiting silicide signatures) for Pd/N,Si-CQDs, and a single-phase face-centered cubic Pt–Pd alloy for Pt_3_Pd_1_/N,Si-CQDs. This highlights the influence of support chemistry and growth kinetics on determining the ultimate phase and architecture of the catalysts.

**Figure 4:**
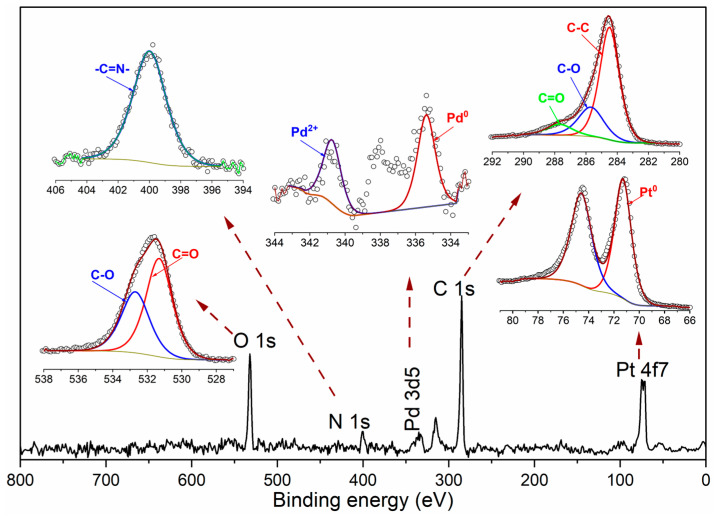
Survey scan and high resolution XPS spectra for Pt_3_Pd_1_/N,Si-CQDs.

Figure 4 presents the survey and high-resolution spectra of the Pt_3_Pd_1_/N,Si-CQDs. The bimetallic catalyst demonstrated electronic modifications that were driven by alloying. In Pt_3_Pd_1_/N,Si-CQDs, the Pt 4f_7_/_2_ peak was observed at approximately 71.29 eV, which was marginally lower than that in Pt/N,Si-CQDs (approximately 71.33 eV).

Conversely, the Pd 3d_5_/_2_ peak exhibited a positive shift to approximately 335.4 eV compared to that of Pd/N,Si-CQDs (approximately 334.9 eV) (Table 1). These opposing shifts, negative for Pt and positive for Pd, indicate a ligand effect associated with net charge transfer from Pd to Pt [32], resulting in a reconfiguration of the surface electronic structure that is known to influence adsorption energetics in electrocatalysis [33]. Deconvolution further revealed a Pd^0^/Pd^2+^ ratio of approximately 61/39 (335.4/337.7 eV), indicating a predominantly metallic Pd surface with a minor oxidized contribution [34].

In the case of Pd/N,Si-CQDs, the XPS surface chemistry was aligned with the XRD phase assignment using a core–shell model. While XRD identified a Pd_9_Si_2_ phase within the bulk, the XPS surface was predominantly characterized by Pd^0^ (≈334.9 eV) and exhibited no detectable Si 2p signals. This perspective is corroborated by Table 2, which indicates that Si was present in the bare N,Si-CQDs (2.29 at.%) but becomes undetectable following metallation (the support spectrum is discussed in Section 3.1). The survey stoichiometry further revealed that the oxygen content was lower in Pd/N,Si-CQDs (16.60 at.%) compared to Pt/N,Si-CQDs (17.23 at.%) and Pt_3_Pd_1_/N,Si-CQDs (20.85 at.%) (Table 2), which is consistent with the formation of silicide occurring in a relatively more reducing environment that consumes a portion of the surface oxygen functionalities.

The heteroatom chemistry of the N,Si-CQDs support was largely retained after metallation. In all samples, the C 1s spectrum resolved into C–C/C=C at approximately 284.5 eV (major), C–N/C–O at approximately 285.7–286.0 eV, and C=O/O–C=O at approximately 287.7–288.0 eV; the O 1s spectrum resolved into C=O at approximately 531.1–531.4 eV and C–O/Si–O–Si at approximately 532.0–532.7 eV. Consistent with Section 3.1, the N 1s envelope remained a single broad peak centered at approximately 399.3–399.9 eV, predominantly composed of pyrrolic/imine-N (–C=N–) with a minor contribution from pyridinic-N, providing plausible Pt–N/Pd–N coordination sites that enhance the metal–support interactions [35,36,37]. The corresponding component atomic percentages for each envelope are presented in Table 1. Collectively, the XPS results corroborate the formation of an alloy with Pd to Pt charge transfer in Pt_3_Pd_1_/N,Si-CQDs, endorse a Pd_9_Si_2_-core/metallic-Pd-shell configuration for Pd/N,Si-CQDs, and affirm the persistence of N/O functionalities that facilitate metal–support bonding in the catalyst. These findings are consistent with and complementary to the structural conclusions derived from the TEM/XRD analyses.

### 3.3. Catalytic Performance of Pt-, Pd-, and Pt_3_Pd_1_/N,Si-CQDs

**Figure 5:**
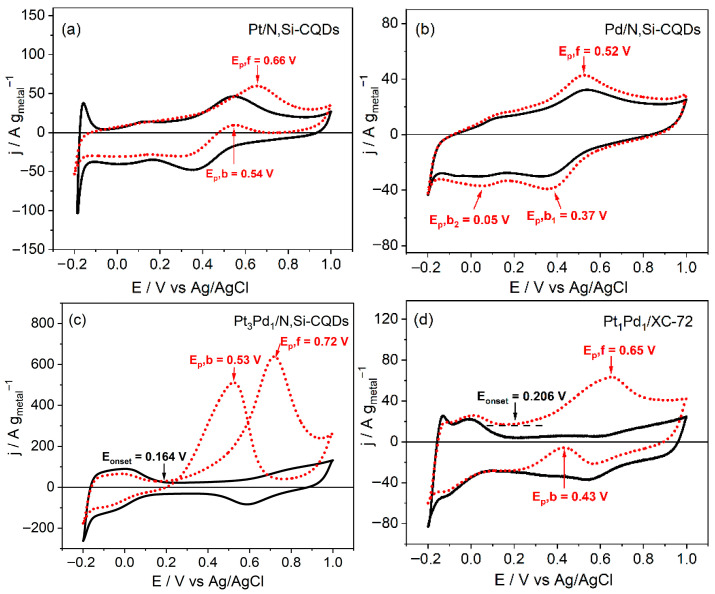
CVs of (**a**) Pt/N,Si–CQDs, (**b**) Pd/N,Si–CQDs, (**c**) Pt_3_Pd_1_/N,Si–CQDs, and (**d**) Pt_1_Pd_1_/XC-72 in 0.5 M H_2_SO_4_ (black/solid) and 0.5 M H_2_SO_4_ + 0.5 M MeOH (red/dotted) at a scan rate of 50 mV s^−1^.

The MOR was assessed using CV in both 0.5 M H_2_SO_4_ and a mixture of 0.5 M H_2_SO_4_ with 0.5 M methanol (MeOH) (Figure 5). In the presence of MeOH, all the catalysts exhibited a characteristic forward scan, whereas the reverse scan varied significantly among the catalysts. The forward-scan peak mass activity of Pt_3_Pd_1_/N,Si–CQDs reached 648.4 A g_metal_^−1^, which was approximately 10 times higher than that of Pt/N,Si–CQDs and the commercial Pt_1_Pd_1_/XC-72 and approximately 15 times higher than that of Pd/N,Si–CQDs.

Table 3 presents a summary of the ECSA (determined in acidic solution), SA, and MA values of the catalysts for the MOR, providing a quantitative comparison of their electrocatalytic performance. When normalized to Pt-ECSA, the specific activity at the forward peak for Pt/N,Si-CQDs was approximately equivalent to that of Pt_3_Pd_1_/N,Si-CQDs (0.427 vs. 0.424 mA/cm^2^), both of which surpassed the performance of the commercial catalyst (0.358 mA/cm^2^) This suggests that the improved mass activity mainly originates from the increased ECSA rather than changes in the intrinsic activity of individual Pt sites. Consequently, alloying maintains the intrinsic activity of Pt sites while significantly enhancing metal utilization through an increased ECSA, providing more accessible active sites for methanol adsorption and oxidation, and thereby leading to an overall improvement in mass activity. Furthermore, the onset potential of Pt_3_Pd_1_/N,Si–CQDs was 0.164 V, which was 42 mV earlier than that of Pt_1_Pd_1_/XC-72 (0.206 V), indicating facilitated MOR kinetics [38]. The ECSA derived from the integration of the H_upd_ region in acid and normalized to Pt, further distinguishes the samples: 181 m^2^ g_Pt_^−1^ for Pt_3_Pd_1_/N,Si–CQDs, compared to 14.3 m^2^ g_Pt_^−1^ for Pt/N,Si–CQDs and 27.7 m^2^ g_Pt_^−1^ for Pt_1_Pd_1_/XC-72 (Table 3). This exceptional ECSA value significantly surpasses those reported for other advanced bimetallic systems, such as Pt-Pd on reduced graphene oxide (81.2 m^2^ g^−1^) [39], and high-performance Pt-Co catalysts (93.8 m^2^ g^−1^) [40], highlighting the superior architecture of the current catalyst. The ECSA value of the bimetallic CQD catalyst aligns with the TEM/XPS evidence of small, homogeneously dispersed alloy nanoparticles anchored by N-donor sites on N,Si–CQDs and Pd-assisted nucleation that enhances the particle number density at a fixed metal loading [41].

The cyclic voltammetry (CV) profiles depicted in Figure 5 offer mechanistic insights into the distinct oxidation behaviors of the Pt-, Pd-, and Pt_3_Pd_1_/N,Si–CQDs catalysts, highlighting the pivotal role of the N,Si-CQDs support. For the monometallic Pt/N,Si–CQDs (Figure 5a), methanol oxidation was initiated at approximately 0.66 V (E_p_,f), with a modest increase in current compared to that in the acidic medium, indicating limited methanol activation on the isolated Pt sites. The backward peak (E_p_,b ≈ 0.54 V) corresponds to the reduction in Pt oxides and the oxidative removal of intermediates, suggesting facile surface regeneration and a relatively clean active surface after the oxidation reaction.

Pd/N,Si–CQDs (Figure 5b) exhibit an earlier onset of oxidation at 0.52 V, approximately 0.14 V lower than that of Pt, thereby demonstrating Pd’s capability to generate OH_ads_ species at lower potentials through water activation. However, the cathodic features at 0.37 and 0.05 V correspond to the reduction in PdO_x_ to Pd^0^ and the formation of PdH_x_, respectively, indicating a slow surface recovery and delayed cleaning of the Pd sites. Although Pd facilitates early oxidation, the accumulation of Pd oxides impedes complete surface regeneration during the reverse scan.

The bimetallic Pt_3_Pd_1_/N,Si–CQDs (Figure 5c) demonstrated significantly enhanced electrochemical properties, as evidenced by a well-defined H_p__u_d and double-layer region (0.15–0.40 V), indicating improved surface accessibility and a larger electrochemically active area. Methanol oxidation was initiated at 0.164 V and increased sharply to a forward peak at 0.72 V. This pronounced anodic response is attributed to the synergistic interaction between Pt and Pd sites. Pt facilitates methanol dehydrogenation, whereas Pd activates water to generate OH_ads_. (Equations (1) and (2)). The co-reaction between Pt–(CO)_ads_ and Pd–(OH)_ads_ enables the rapid oxidative removal of CO_ads_ intermediates (Equation (3)).

Pt + CH_3_OH → Pt–(CO)_ads_ + 4H^+^ + 4e^−^ (1)

Pd + H_2_O → Pd–(OH)_ads_+ H^+^ + e^−^ (2)

Pt–(CO)_ads_ + Pd–(OH)_ads_ → Pt + Pd + CO_2_ + H^+^ + e^−^ (3)

The backward peak (E_p_,b ≈ 0.53–0.54 V) corresponds to that of Pt/N,Si–CQDs, suggesting a comparable intrinsic specific activity but a significantly higher mass activity due to its larger ECSA.

The enhanced performance of Pt_3_Pd_1_/N,Si–CQDs can be attributed to the robust metal–support interactions between the alloy nanoparticles and N,Si-CQDs matrix. Nitrogen dopants offer electron-rich anchoring sites that stabilize the metal nanoparticles, whereas Si–O functional groups strengthen the interfacial bonding and facilitate uniform dispersion. This dual chemical–electronic coupling improves the charge redistribution between Pt, Pd, and the support, thereby amplifying both the bifunctional and ligand effects. Consequently, the alloy structure exhibits numerous Pt–Pd interfacial sites that continuously generate OH_ads_ species and oxidize CO_ads_ at lower overpotential.

The commercial Pt_1_Pd_1_/XC-72 catalyst (Figure 5d) exhibited a forward peak at 0.65 V, similar to that of Pt, but demonstrated a delayed backward feature at 0.43 V. This observation suggests a slower oxidation process for the adsorbed intermediates and reduced tolerance to CO. Although a double-layer region was discernible, the overall anodic response was inferior to that of Pt_3_Pd_1_/N,Si–CQDs. This underscores the significance of the naturally heteroatom-enriched CQDs support in optimizing the active-site utilization and ensuring durable metal dispersion.

These findings collectively demonstrate that while Pd facilitates the initial formation of OH_ads_ and Pt offers high intrinsic activity, their integration on the N,Si-CQD support results in a cooperative bifunctional–ligand mechanism. This mechanism is characterized by enhanced charge transfer, an increased electrochemical surface area (ECSA), and superior resistance to CO poisoning.

In acidic media, the reported forward-peak specific activity values typically range from approximately 0.4 to 1.7 mA cm^−2^, notwithstanding variations in the electrolyte composition, methanol concentration, and scan rate [42,43]. The value obtained in this study (≈0.42 mA cm^−2^) falls within this range and surpasses that of the commercial reference catalyst. Regarding mass activity, Pt_3_Pd_1_/N,Si–CQDs achieved 648 A g_metal_^−1^ (i.e., 766 A g_Pt_^−1^), surpassing typical commercial Pt/C catalysts surpassing typical commercial Pt/C catalysts (≈100–400 A g_Pt_^−1^) [44,45] and comparable to several state-of-the-art Pt-based catalysts (≈650–800 A g_pt_^−1^) [46,47,48,49]; by contrast, advanced Pt–Pd nanowires can exceed 2000 A g_pt_^−1^ [50].

The scan rate experiments demonstrated that j_F_ ∝ v^1/2^ for all catalysts, exhibiting excellent linearity (R^2^ = 0.979–0.999), thereby confirming diffusion-controlled methanol transport during the forward scan (Figure S2) [51]. The j_F_ ∝ v^1/2^ slope for Pt_3_Pd_1_/N,Si–CQDs (56.35) significantly surpassed those of the other electrodes (8.37–10.39), indicating a substantially higher density of accessible active sites under the identical conditions (Figure S3). The behavior observed in the reverse scan is diagnostic for Pt/N,Si–CQDs, Pd/N,Si–CQDs, and Pt_1_Pd_1_/XC-72, the current near the nominal backward peak was below zero (cathodic), resulting in j_F_/j_B_ ≫ 1. In Pd/N,Si–CQDs, two cathodic features are discernible at ≈0.37 V and ≈0.05 V (Figure 5b), attributable to from PdO_x_ to Pd^0^ reduction and hydrogen adsorption/absorption (PdH_x_ formation), respectively [52]; these Pd-specific processes predominate in the reverse scan and inhibit the oxidative removal of intermediates. This behavior is entirely consistent with a Pd-rich surface, as evidenced by XPS, and aligns with the Pd_9_Si_2_-core/metallic-Pd-shell model proposed in Section 3.2.

**Figure 6:**
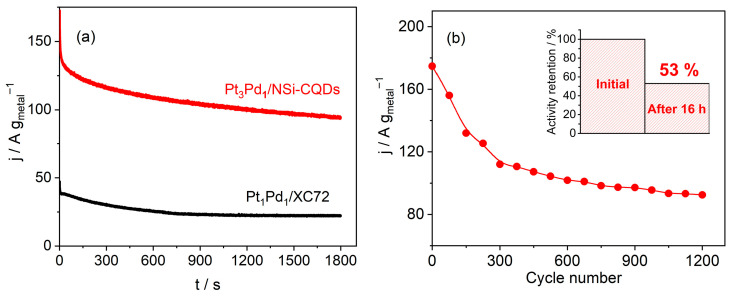
(**a**) Chronoamperogram of Pt_3_Pd_1_/N,Si–CQDs (red), and commercial Pt_1_Pd_1_/XC-72 (black) recorded at 0.50 V for 1800 s in 0.5 M H_2_SO_4_ + 0.5 M MeOH. (**b**) ADT of Pt_3_Pd_1_/N,Si–CQDs by cycling −0.20–1.00 V; mass activity at 0.50 V plotted against cycle number. Inset: Activity retention after 1200 cycles (~16 h).

The commercial Pt_1_Pd_1_/XC-72 exhibited a marked increase in the backward feature with the scan rate, indicative of the adsorption-controlled oxidation of surface-accumulated intermediates (e.g., CO), whereas the growth of the reverse feature was comparatively subdued for Pt_3_Pd_1_/N,Si–CQDs, indicating superior poisoning tolerance. The observed kinetic signatures are consistent with the XPS results presented in Section 3.2, indicating the presence of a Pt–Pd alloy characterized by electron transfer from Pd to Pt, known as the ligand effect. Additionally, the presence of a Pd(II) fraction capable of generating OH_ads_ at lower potentials facilitates continuous CO removal through a bifunctional mechanism and weakens Pt–CO bonding. Two empirical observations support this hypothesis. First, within the MeOH-containing electrolyte, the red CV traces did not occupy the H_upd_ region for any catalyst. This is anticipated because, at low potentials, the surface is covered by H_upd_ (and, on Pd, by absorbed H in PdH_x_) [49], which impedes the adsorption and dehydrogenation of MeOH. Consequently, the MOR only progresses after H_upd_ desorbs with increasing potential, thereby justifying the ECSA determination in MeOH-free acid. Second, potentiostatic tests underscored the steady-state advantage of the bimetallic CQD catalyst during chronoamperometry at 0.50 V for 1800 s in 0.5 M H_2_SO_4_ + 0.5 M MeOH (Figure 6a), Pt_3_Pd_1_/N,Si–CQDs sustained a significantly higher mass current than the benchmark; at 1800 s, it delivered approximately 95 A g_metal_^−1^ compared to approximately 23 A g_metal_^−1^ for Pt_1_Pd_1_/XC-72, which was approximately four times higher.

Under an accelerated durability protocol (−0.20–1.00 V, 1200 cycles ≈ 16 h; Figure 6b), the mass activity of Pt_3_Pd_1_/N,Si–CQDs at 0.50 V declined rapidly over the initial ~300 cycles (from 175.8 to 111.6 A g_metal_^−1^) and then entered a slow-decay plateau, reaching 92.7 A g_metal_^−1^, retaining 53% of the initial value. The two-stage profile suggests an initial loss of less-stable particles, followed by the persistence of well-anchored alloyed nanoparticles strongly coupled to the N,Si–CQDs support, reflecting the structural evolution typically observed in alloy catalysts during ADT. This degradation behavior agrees with previously reported trends for Pt and Pt-based catalysts [53,54,55].

In summary, Pt_3_Pd_1_/N,Si–CQDs exhibited an earlier onset, more than ten times higher peak mass activity, an exceptionally large ECSA, enhanced resistance to CO-type poisoning, and robust steady-state and ADT performance (Figure 5 and Figure 6, Table 3). These characteristics are fully consistent with the alloyed structure and strong metal–support coupling established in Section 3.2.

## 4. Conclusions

A high-performance Pt_3_Pd_1_/N,Si–CQDs electrocatalyst was successfully synthesized via a hydrothermal route using stinging nettle (*Urtica dioica* L.) as a single sustainable biomass precursor. The biogenic Si and N species within the CQD matrix acted as intrinsic nucleation and anchoring sites, enabling the formation of uniformly dispersed Pt_3_Pd_1_ nanoalloys with strong metal–support interactions.

The resulting catalyst achieved an ECSA of 181 m^2^ g_Pt_^−1^ and a peak mass activity of 648.4 A g_metal_^−1^, approximately tenfold higher than that of the commercial Pt_1_Pd_1_/XC-72 catalyst, with a low onset potential (0.164 V) and 53% activity retention after a 16 h ADT. These findings highlight the combined benefits of alloying and heteroatom-functionalized CQD supports for enhanced MOR activity and durability of the catalyst.

Overall, this study proposes a sustainable and effective approach to catalyst design, demonstrating that naturally occurring heteroatoms in biomass can serve as active structural components to facilitate metal nucleation and enhance the electrocatalytic performance of DMFCs.

## Figures and Tables

**Table 1:** Binding energies (BEs) and atomic percentages (at.%, in parentheses) from high-resolution XPS of the catalysts. The listed binding energies are consistent with the reported literature ranges [19,26,27,28,30,31,32,33,34,35].

Element	BEs (at.%)				Identification
	S0	S1	S2	S3	
Pt 4f_7/2_	-	71.33 (100)	-	71.29 (100)	Pt^0^
Pd 3d_5/2_	-	-	334.87 (100)	335.38 (61) 337.70 (39)	Pd^0^ Pd^2+^
O 1s	531.03 (83) 532.03 (17)	531.44 (69) 532.83 (31)	531.11 (40) 532.45 (60)	531.31 (60) 532.69 (40)	C=O C-O/Si-O-Si
N 1s	399.33 (100)	399.86 (100)	399.69 (100)	399.99 (100)	-C=N-
C 1s	284.22 (76) 285.77 (10) 287.75 (14)	284.50 (76) 285.81 (18) 287.74 (6)	284.50 (69) 286.06 (26) 288.04 (5)	284.50 (68) 285.68 (23) 287.68 (9)	C-C/C=C C=O/C-N C=O/O-C=O

S0: N,Si-CQDs; S1: Pt/N,Si-CQDs; S2: Pd/N,Si-CQDs; S3: Pt_3_Pd_1_/N,Si-CQDs.

**Table 2:** Survey XPS–derived surface atomic percentages (at.%) for catalysts.

Sample ^a^	Surface Atomic Percentages (at.%)					
	Pt	Pd	C	O	N	Si
S0 ^b^	-	-	66.42	21.37	7.24	2.29
S1	1.70	-	75.48	17.23	5.59	n.d.
S2	-	1.63	76.24	16.60	5.53	n.d.
S3	2.61	0.23	70.74	20.85	5.56	n.d.

^a^ S0: N,Si-CQDs; S1: Pt/N,Si-CQDs; S2: Pd/N,Si-CQDs; S3: Pt_3_Pd_1_/N,Si-CQDs. ^b^ Total atomic percentage less than 100% due to omission of Ca and K. n.d. = not detected (below the instrumental detection limit).

**Table 3:** Electrochemical characterization for MOR.

Catalysts ^a^	E_onset_ (V)	ECSA (m^2^ g _Pt_^−1^)	MA (A g_metal_^−1^)	SA (mA/cm^2^_Pt_)	Activity ^b^ (A g_catalyst_^−1^)
S1	-	14.3	61.05	0.427	3.05
S2	-	-	43.35	-	2.17
S3	0.164	181.0	648.39	0.424	32.42
S4	0.206	27.7	64.05	0.358	6.41

^a^ S1: Pt/N,Si-CQDs; S2: Pd/N,Si-CQDs; S3: Pt_3_Pd_1_/N,Si-CQDs; S4: Pt_1_Pd_1_/XC72. ^b^ Normalized to total catalyst mass (support + metal).

## Data Availability

Data is contained within the article or Supplementary Materials.

## Supplementary Materials

The following supporting information can be downloaded at: https://www.mdpi.com/article/10.3390/nano15201561/s1, Figure S1: Schematic illustration of the synthesized catalysts supported on N,Si-CQDs; Figure S2: Cyclic voltammograms at different scan rates in 0.5 M H2SO4 + 0.5 M MeOH for (a) Pt/N,Si–CQDs, (b) Pd/N,Si–CQDs, (c) Pt3Pd1/N,Si–CQDs, and (d) Pt1Pd1/N,Si–CQDs; Figure S3: Mass activity (jF) as a function of v1/2 for (a) Pt/N,Si–CQDs, (b) Pd/N,Si–CQDs, (c) Pt3Pd1/N,Si–CQDs, and (d) Pt1Pd1/XC-72 in 0.5 M H2SO4 + 0.5 M MeOH. Linear fits are depicted with the slope and R2 for each fit provided.

## Abbreviations

The following abbreviations are used in this manuscript:

DMFCs	Direct methanol fuel cells
MOR	Methanol oxidation reaction
ORR	Oxygen reduction reaction
CO	Carbon monoxide
CQDs	Carbon quantum dots
MCE	Mixed cellulose esters
UV-Vis	Ultraviolet visible
PL	Photoluminescence
EEM	Excitation emission matrix
ATR-FTIR	Attenuated total reflection-Fourier transform infrared
XRD	X-ray diffraction
ICDD	International center for diffraction data
JCPDS	Joint committee on powder diffraction standards
XPS	X-ray photoelectron spectroscopy
TEM	Transmission electron microscopy
NIH	National Institutes of Health
GC	Glassy carbon
CV	Cyclic voltammetry
MA	Mass activity
SA	Specific activity
CA	Chronoamperometry
ADT	Accelerated durability test
ECSA	Electrochemically active surface area
MeOH	Methanol
