# Supplementary material for: Harnessing Biogenic Silica: Nanoarchitected Pt3Pd1 on Nettle-Derived N,Si-CQDs for High-Performance Methanol Electrooxidation
Source: Nanomaterials (Basel). 2025 Oct 14;15(20):1561. doi: 10.3390/nano15201561 (PMC12566391; doi:10.3390/nano15201561)
Supplement: Supplementary file 1 [file nanomaterials-15-01561-s001.zip › nanomaterials-3905399-supplementary.pdf]

Supporting Information

Harnessing Biogenic Silica: Nanoarchitected Pt_3Pd_1 on Nettle-Derived N,Si-CQDs for High-Performance Methanol Electrooxidation

Seden Beyhan [1,*]

[1]Department of Chemistry, Faculty of Science and Letters, Istanbul Technical University, 34469 Maslak, Istanbul, Turkey
* Correspondence: beyhanse@itu.edu.tr

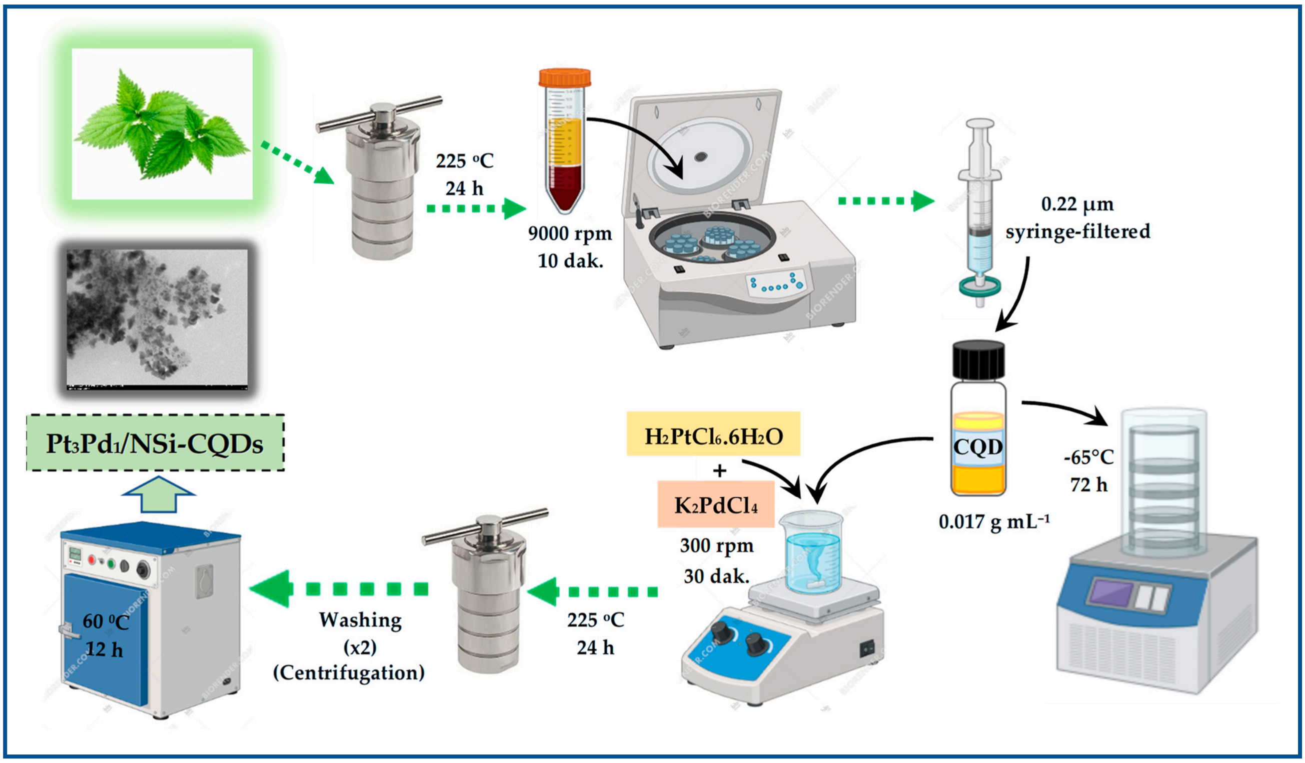

Figure S1. Schematic illustration of the synthesized catalysts supported on N,Si-CQDs.

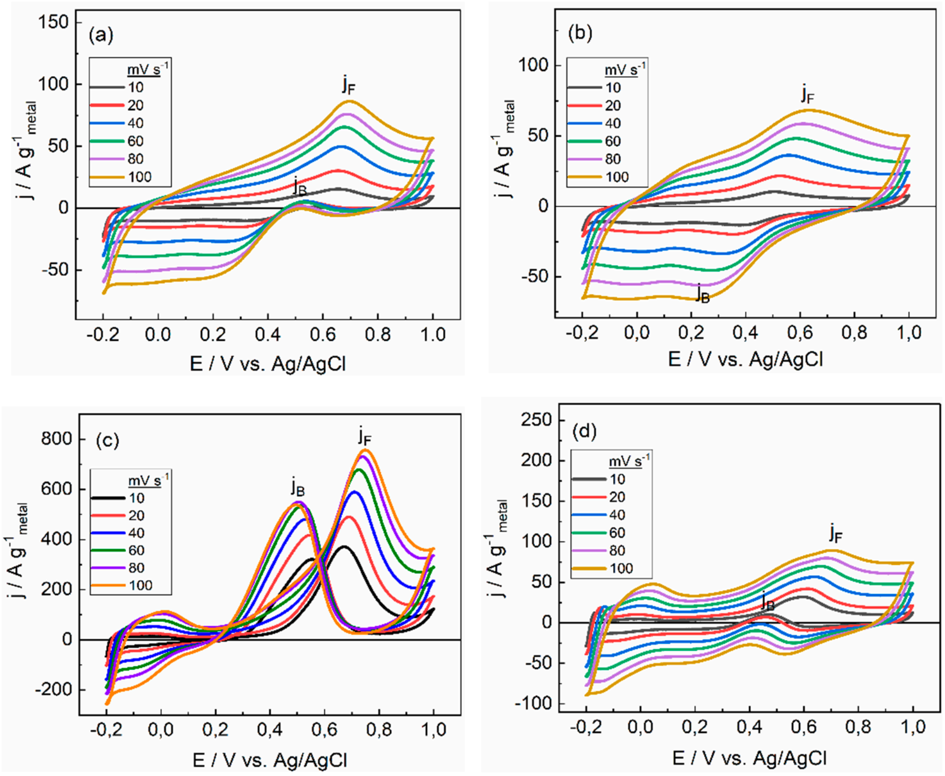

Figure S2. Cyclic voltammograms at different scan rates in 0.5 M H_2SO_4 + 0.5 M MeOH for (a) Pt/N,Si–CQDs, (b) Pd/N,Si–CQDs, (c) Pt_3Pd_1/N,Si–CQDs, and (d) Pt_1Pd_1/N,Si–CQDs.

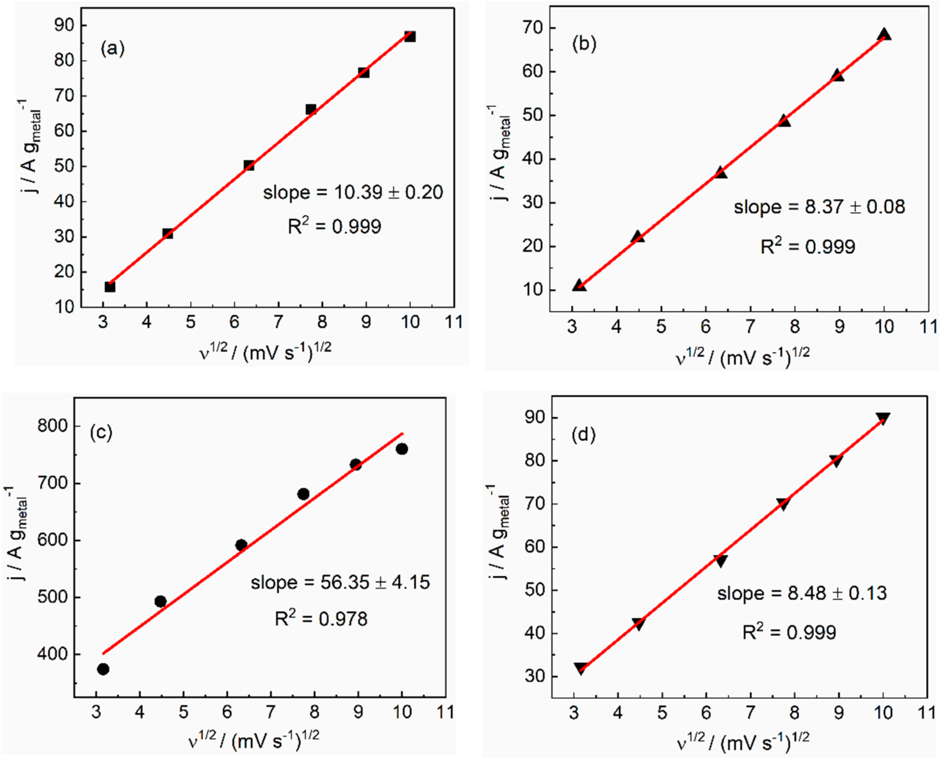

Figure S3. Mass activity (j_F) as a function of $v^{1/2}$ for (a) Pt/N,Si–CQDs, (b) Pd/N,Si–CQDs, (c) Pt_3Pd_1/N,Si–CQDs, and (d) Pt_1Pd_1/XC-72 in 0.5 M H_2SO_4 + 0.5 M MeOH. Linear fits are depicted with the slope and R^2 for each fit provided.
